# Shared genetic correlations between kidney diseases and sepsis

**DOI:** 10.3389/fendo.2024.1396041

**Published:** 2024-07-17

**Authors:** Tianlong Zhang, Ying Cui, Siyi Jiang, Lu Jiang, Lijun Song, Lei Huang, Yong Li, Jiali Yao, Min Li

**Affiliations:** ^1^ Department of Critical Care Medicine, The Fourth Affiliated Hospital of School of Medicine, and International School of Medicine, International Institutes of Medicine, Zhejiang University, Yiwu, China; ^2^ Department of Critical Care Medicine, Jinhua Hospital Affiliated to Zhejiang University, Jinhua, Zhejiang, China

**Keywords:** sepsis, kidney, genetic correlation, blood urea nitrogen, creatinine, urinary albumin-to-creatinine ratio, urate, kidney stones

## Abstract

**Background:**

Clinical studies have indicated a comorbidity between sepsis and kidney diseases. Individuals with specific mutations that predispose them to kidney conditions are also at an elevated risk for developing sepsis, and vice versa. This suggests a potential shared genetic etiology that has not been fully elucidated.

**Methods:**

Summary statistics data on exposure and outcomes were obtained from genome-wide association meta-analysis studies. We utilized these data to assess genetic correlations, employing a pleiotropy analysis method under the composite null hypothesis to identify pleiotropic loci. After mapping the loci to their corresponding genes, we conducted pathway analysis using Generalized Gene-Set Analysis of GWAS Data (MAGMA). Additionally, we utilized MAGMA gene-test and eQTL information (whole blood tissue) for further determination of gene involvement. Further investigation involved stratified LD score regression, using diverse immune cell data, to study the enrichment of SNP heritability in kidney-related diseases and sepsis. Furthermore, we employed Mendelian Randomization (MR) analysis to investigate the causality between kidney diseases and sepsis.

**Results:**

In our genetic correlation analysis, we identified significant correlations among BUN, creatinine, UACR, serum urate, kidney stones, and sepsis. The PLACO analysis method identified 24 pleiotropic loci, pinpointing a total of 28 nearby genes. MAGMA gene-set enrichment analysis revealed a total of 50 pathways, and tissue-specific analysis indicated significant enrichment of five pairs of pleiotropic results in kidney tissue. MAGMA gene test and eQTL information (whole blood tissue) identified 33 and 76 pleiotropic genes, respectively. Notably, genes *PPP2R3A* for BUN, *VAMP8* for UACR, *DOCK7* for creatinine, and *HIBADH* for kidney stones were identified as shared risk genes by all three methods. In a series of immune cell-type-specific enrichment analyses of pleiotropy, we identified a total of 37 immune cells. However, MR analysis did not reveal any causal relationships among them.

**Conclusions:**

This study lays the groundwork for shared etiological factors between kidney and sepsis. The confirmed pleiotropic loci, shared pathogenic genes, and enriched pathways and immune cells have enhanced our understanding of the multifaceted relationships among these diseases. This provides insights for early disease intervention and effective treatment, paving the way for further research in this field.

## 1 Background

Sepsis is a life-threatening disease that endangers human lives and results in multiple organ dysfunction (1). It remains one of the leading causes of morbidity and mortality worldwide, highlighting the critical need to understand its impact on public health (2). In 2017 alone, an estimated 48.9 million cases of sepsis occurred globally, resulting in 11 million deaths, accounting for nearly 20% of all global deaths (3). Due to limited treatment options, identifying the underlying mechanisms of sepsis is essential for developing novel therapeutic strategies.

Sepsis presents with a heterogeneous range of clinical symptoms (4), with renal dysfunction being a frequent complication. Early stages of various kidney diseases often manifest as renal impairment, characterized by changes in blood creatinine, blood urea nitrogen (BUN), urinary albumin-to-creatinine ratio (UACR), uric acid (UA), estimated glomerular filtration rate (eGFR), and other relevant indicators. For instance, elevated blood creatinine levels are frequently observed in sepsis patients, a phenomenon also characteristic of acute kidney injury and chronic kidney disease (5). Similarly, changes in BUN, UACR, and UA have been closely linked to sepsis (6–8). Additionally, studies suggest an association between stone formation and adverse outcomes like sepsis (9). The connections between IgA nephropathy, diabetic nephropathy, renal tumors, and sepsis also require further elucidation. The shared genetic underpinnings of these associations remain unexplored. Therefore, a systematic analysis is crucial to investigate the presence of common pleiotropic risk variants between kidney-related diseases or relevant indicators and sepsis, along with the potential involvement of specific biological pathways and shared risk genes.

The rise of genome-wide association studies (GWAS) in recent years has been instrumental. GWAS have become a powerful tool for identifying a wide range of genetic variations associated with complex diseases and elucidating genes related to the occurrence, progression, and treatment of diseases (10). In our GWAS study, patients with acute kidney injury and chronic kidney disease predominantly exhibited elevated indicators such as creatinine and eGFR. Therefore, we used these kidney-related indicators as proxies for the two conditions. Utilizing large-scale GWAS summary data, we conducted a genome-wide analysis for nine kidney-related diseases or indicators (BUN, UACR, UA, eGFR, creatine, kidney neoplasm, kidney stone, IgA nephropathy, and diabetic nephropathy) and sepsis. We employed various genetic methods, including pleiotropy analysis for significant results, to systematically explore pleiotropic associations at both the gene and pathway levels. This analysis aimed to uncover potential shared genetic etiology. Additionally, we conducted gene set enrichment analysis and immune cell enrichment analysis to discover potential correlations. Finally, Mendelian randomization (MR) analysis was employed to explore causal relationships among these factors.

## 2 Methods

### 2.1 Study design

**Figure 1:**
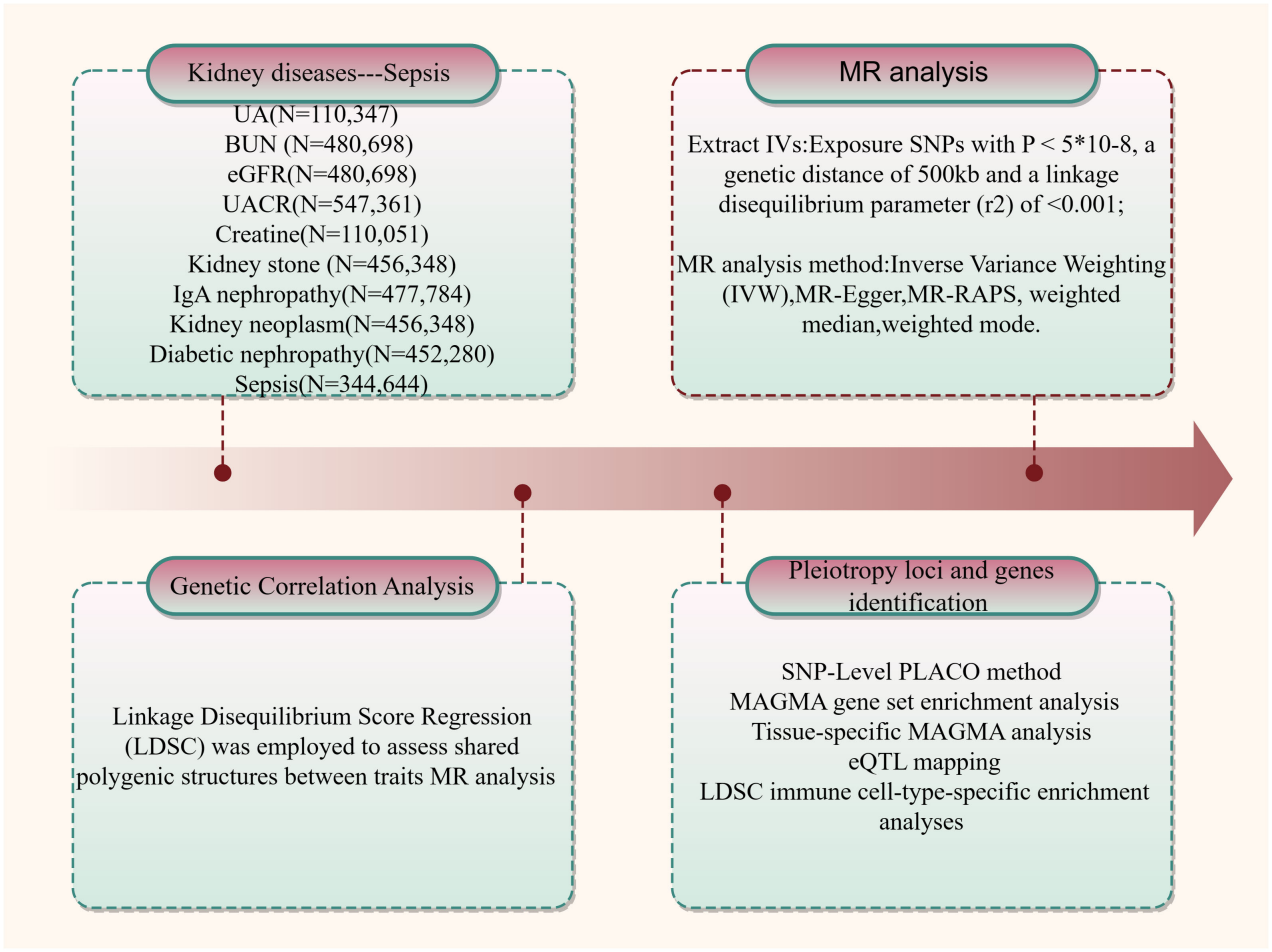
An overview of the study design.

This study investigates the potential genetic associations between kidney disease or relevant kidney function indicators and sepsis. A schematic representation of the study design is provided in **Figure 1**.

### 2.2 GWAS data sources

In our analyses, we investigated the genetic associations between the following traits: BUN (N=480,698), UACR (N=547,361), UA (N=110,347), eGFR (N=480,698), creatinine (N=110,051), kidney neoplasm (N=456,348), kidney stone disease (N=456,348), IgA nephropathy (N=477,784), diabetic nephropathy (N=452,280), and sepsis (N=344,644) (11–15). Details regarding the data sources for the GWAS employed in this analysis are presented in **Supplementary Table S1**. All studies included in these consortia were approved by local research ethics committees and institutional review boards, and written informed consent was obtained from all participants.

### 2.3 Statistical analyses

#### 2.3.1 Genetic correlation analysis

Linkage Disequilibrium Score Regression (LDSC) was employed to assess shared polygenic structures between traits, with LD scores calculated from European ancestry samples of the 1000 Genomes Project serving as the reference group (1). Stringent quality control measures were implemented for SNPs: (i) exclusion of non-biallelic SNPs and those with ambiguous allele chains; (ii) removal of SNPs lacking rs labels, duplicates, or those not included in the 1000 Genomes Project, with alleles mismatching that dataset, or located within the major histocompatibility complex region (chr6: 28.5–33.5Mb); (v) retention of SNPs with a minor allele frequency (MAF) > 0.01. Given the distinct origins of kidney-related diseases and sepsis, there was limited sample overlap between them. Finally, we preserved pertinent information for each SNP, such as effect size, standard error, effect allele, and P-value, for further analysis.

Stratified LD score regression was applied to immune cell data to study whether specific cell types harbor a greater proportion of the genetic variation (SNP heritability) underlying kidney-related traits and sepsis. We aimed to identify immune cell types with significant enrichment of SNP heritability. Data on 292 immune cell types from the ImmGen consortium (including B cells, gamma delta T cells, alpha beta T cells, innate lymphocytes, myeloid cells, stromal cells, and stem cells) were used (16). Following adjustment for baseline models and all gene sets, significance of SNP heritability enrichment estimates in each tissue and cell type was assessed using p-values from regression coefficients z-scores.

#### 2.3.2 Analysis of pleiotropy under the composite null hypothesis

SNP-Level PLACO leverages summary-level genotype-phenotype association statistics to investigate pleiotropic loci among complex traits (17). We computed the squared Z-scores for each variant, excluding SNPs with extremely high Z^2^ (>80). Additionally, considering the potential correlation between kidney-related diseases and sepsis, we estimated the correlation matrix of Z-scores. Subsequently, a Horizontal α-level intersection-union test (IUT) method was employed to test the hypothesis of no pleiotropy. The final p-value from the IUT test represents the strongest evidence against the null hypothesis.To explore the shared biological mechanisms of these pleiotropic loci identified by PLACO, we further mapped them to nearby genes. MAGMA analysis (18) was conducted on genes located at or overlapping with the pleiotropic loci, utilizing both PLACO outputs and single-trait GWAS results. A significance level of P< 0.05/N_genes_ = 3E-06 was applied. To identify potential functional annotations beyond the immediate risk loci, we utilized functional maps and annotations from the Functional Mapping and Annotation of Genome-Wide Association Studies (FUMA) (19). Mapping and Annotation of Genome-Wide Association Studies (FUMA) (19). Additionally, enrichment analysis of mapped genes was performed using pathways from the Molecular Signatures Database (MSigDB) (20). Additionally, we employed eQTL mapping, which identifies genes whose expression is influenced by genetic variants, to explore associations beyond the risk loci themselves. Tissue data originated from the GTEx V8 eQTL summary data for whole blood tissue (21).

#### 2.3.3 MR analysis

We used PLINK’s clumping program (22) to identify potential causal variants among loci significantly associated with exposure (P< 5×10^-8^). These loci were treated as instrumental variables (IVs) in downstream analyses. All IVs underwent linkage disequilibrium (LD) clumping (r^2 ^= 0.001; distance = 5,00 kb) to mitigate the influence of correlated SNPs. To ensure the strength of the IVs, we computed the R^2^ and F-statistic $\left(F=\left(\frac{N-1-k}{k}\right)\left(\frac{R^{2}}{1-R^{2}}\right)\right)$ , for each IV, where R^2^ represents the proportion of variance explained by the genetic instrument, N is the effective sample size of GWAS, and k is the number of SNPs (23).

Mendelian randomization primarily relies on the Inverse Variance Weighting (IVW) approach, but this method hinges on three key assumptions. First, the genetic variants (instrumental variables) must be strongly associated with the exposure of interest. Second, these variants should not be influenced by confounding factors that affect both the exposure and the outcome. Finally, the variants’ effect on the outcome must solely act through the exposure, with no independent pathways. To assess potential violations of these assumptions, we conducted several sensitivity analyses. Firstly, both the IVW and MR-Egger methods employed the Q-test to detect heterogeneity in associations among individual instrumental variables. This test helps identify potential violations of the core assumptions. Secondly, MR-Egger was used to estimate horizontal pleiotropy based on its intercept, ensuring the genetic variants are independent influences on the exposure and outcome (24). To further strengthen the results’ stability and robustness, we performed supplementary analyses using additional MR methods with varying modeling assumptions and advantages, such as the weighted median and weighted mode.

All statistical analyses were conducted using R version 4.2.3. MR analyses were performed using the MendelianRandomization package (25).

## 3 Results

### 3.1 Genetic correlation analysis

**Figure 2:**
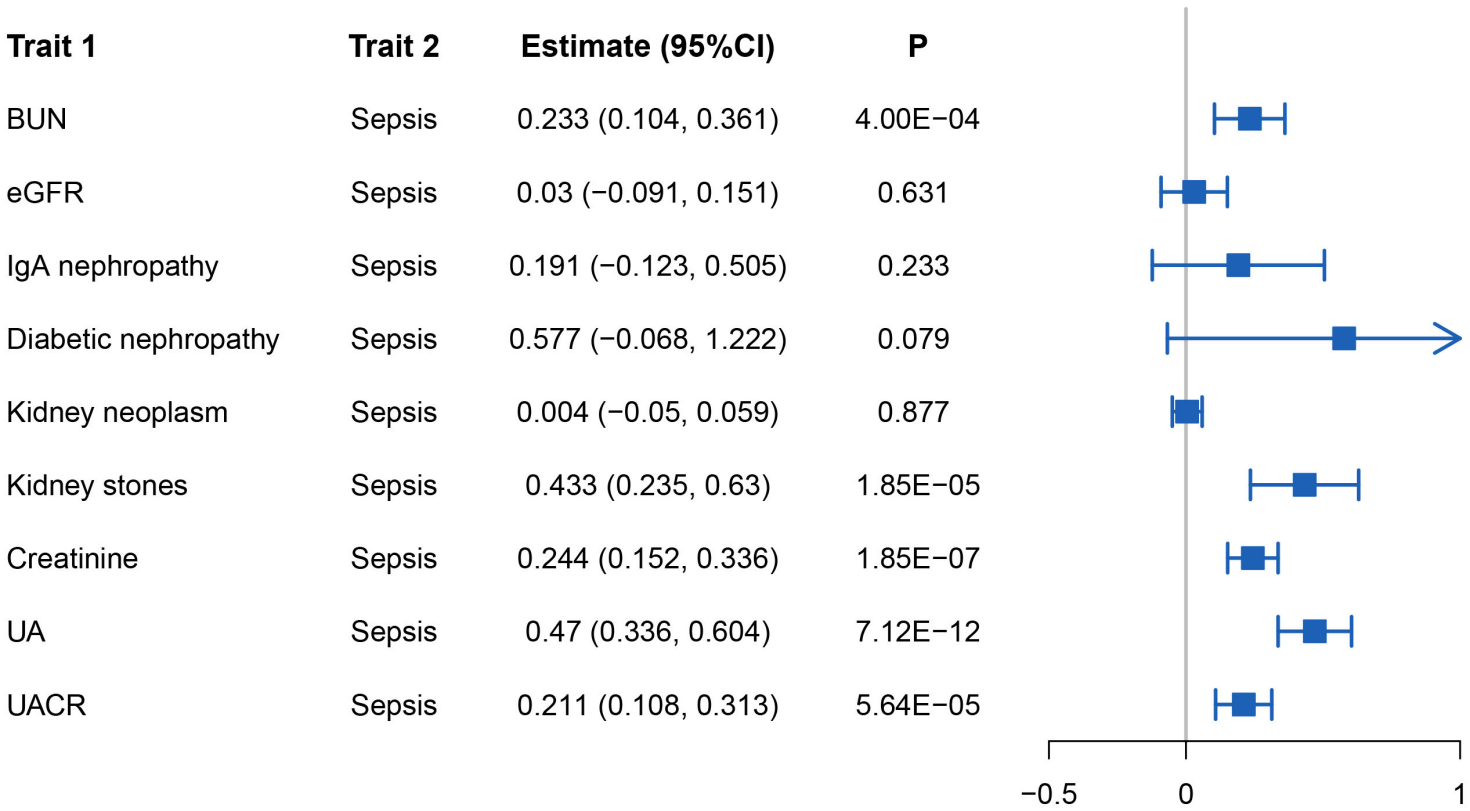
The results of the genetic correlation analysis.

Genetic correlation analysis revealed significant genetic correlations between sepsis and BUN (*r_g_* = 0.233, *P* = 4×10^-4^), kidney stone (*r_g_* = 0.433, *P* = 1.85×10^-5^), creatinine (*r_g_* =0.244, *P* = 1.85×10^-7^), UA (*r_g_* = 0.470, *P* = 7.12×10^-12^), and UACR (*r_g_* = 0.211, *P* = 5.64×10^-5^). To account for multiple testing, we applied Bonferroni correction (0.05/9 = 0.006). Notably, all the aforementioned associations with sepsis remained statistically significant after correction. **Figure 2** illustrates the results of the genetic correlation analysis.

### 3.2 Analysis of PLACO

**Figure 3:**
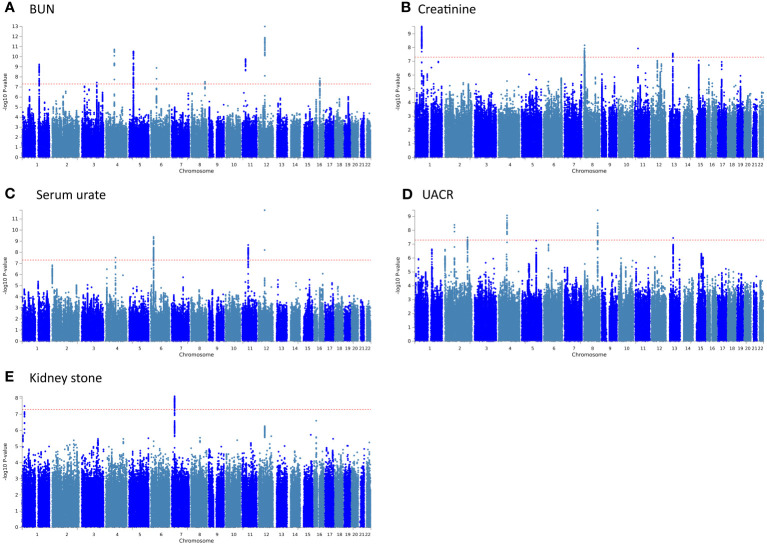
The Manhattan plots for pleiotropic loci in 5 pairs of results. **(A)** BUN and Sepsis; **(B)** Creatinine and Sepsis; **(C)** Serum urate and Sepsis; **(D)** UACR and Sepsis; **(E)** Kidney stone and Sepsis.

PLACO pleiotropy analysis was performed on the five trait pairs exhibiting significant correlations (**Figure 3**). Details of the identified pleiotropic loci are provided in **Table 1** and **Supplementary Table S3**. The analysis collectively identified 24 genomic regions. Notably, the 4q21.1 locus was concurrently associated with both BUN and UACR. Lead SNPs, rs10008637 and rs17319721 (both introns), reside within the SHROOM3 gene. Similarly, the 11p14.1 locus associated with BUN and Creatinine harbors lead SNPs rs685270 and rs963837 (intergenic) near genes *RP5–1024C24.1* and *DCDC1*. The 12q13.3 locus linked to BUN and Serum urate contains lead SNPs rs540730 and rs1106766 (introns) within the *R3HDM2* gene. Finally, the 13q14.3 locus associated with Creatinine and UACR harbors lead SNPs rs1327653 and rs1239707 (intergenic) with currently unidentified associated genes.

**Table 1:** Pleiotropic loci (p-value< 5E-8) identified among the 5 significant result pairs.

Trait pairs	GenomicLocus	Locus boundary	LeadSNPs	P	Nearby gene
BUN&Sepsis	1q22	1:155013708–156005431	rs11264313	5.84E-10	EFNA3, Y_RNA
BUN&Sepsis	3q22.3	3:135462536–136816214	rs6793835	3.61E-08	PPP2R3A
BUN&Sepsis	4q21.1	4:76814640–77693256	rs17319721	1.87E-11	SHROOM3
BUN&Sepsis	5p13.1	5:39758532–41267218	rs4509070	2.96E-11	PTGER4, TTC33
BUN&Sepsis	6p12.3	6:50526289–51370132	rs283561	1.29E-09	FTH1P5, RP3–437C15.2
BUN&Sepsis	8q24.21	8:127485569–127630223	rs73705665	3.00E-08	RP11–103H7.1
BUN&Sepsis	11p14.1	11:30312421–31746547	r685270	1.72E-10	RP5–1024C24.1, DCDC1
BUN&Sepsis	12q13.3	12:57272547–58422642	rs540730	9.89E-14	R3HDM2
BUN&Sepsis	16q12.2	16:53579222–53848561	rs56094641	1.41E-08	FTO
Creatinine&Sepsis	1p31.3	1:62833553–63372868	rs1167998	2.92E-10	DOCK7
Creatinine&Sepsis	8p23.1	8:7626198–10383226	rs6980728	6.82E-09	RP11–375N15.1, RP11–375N15.2
Creatinine&Sepsis	11p14.1	11:30630607–31388640	rs963837	1.17E-08	RP5–1024C24.1, DCDC1
Creatinine&Sepsis	13q14.3	13:50423680–51423481	rs1327653	2.70E-08	NA, NA
UACR&Sepsis	2p11.2	2:85680453–85918616	rs35565292	4.08E-09	VAMP8
UACR&Sepsis	2q33.2	2:202869199–204445015	rs140750546	3.22E-08	RP11–544H14.1, NBEAL1
UACR&Sepsis	4q21.1	4:76814640–77693256	rs10008637	8.44E-10	SHROOM3
UACR&Sepsis	8q24.13	8:126447308–126533955	rs28601761	3.52E-10	RP11–136O12.2
UACR&Sepsis	13q14.3	13:50550018–51423481	rs1239707	3.53E-08	NA, NA
Serum urate&Sepsis	4q22.1	4:88479645–89136066	rs2169611	3.02E-08	HSP90AB3P, SPP1
Serum urate&Sepsis	6p22.2	6:25452783–26770791	rs12209856	4.08E-10	SLC17A1
Serum urate&Sepsis	11q12.1	11:55600975–57092768	rs617212	2.18E-09	OR5G3, AP000479.1
Serum urate&Sepsis	12q13.3	12:57256380–58428763	rs1106766	1.62E-12	R3HDM2
Kidney stone&Sepsis	1p36.12	1:21829792–22351947	rs1256326	3.13E-08	ALPL, RP11–63N8.3
Kidney stone&Sepsis	7p15.2	7:27355114–28037674	rs1404278	7.68E-09	HIBADH

The QQ plot (**Supplementary Figure S1**) did not exhibit premature deviation from the reference line, indicating no genomic inflation. Essential information for each genomic risk locus is provided in **Supplementary Figures S2**, **S3**. **Supplementary Figure S3** explores the functional consequences of cis-acting pleiotropic SNPs on genes. In the association analysis involving BUN, UACR, and kidney stones, these pleiotropic SNPs predominantly affect introns. For serum urate, they mainly impact the intergenic regions, while their influence on creatinine is relatively minor.

**Figure 4:**
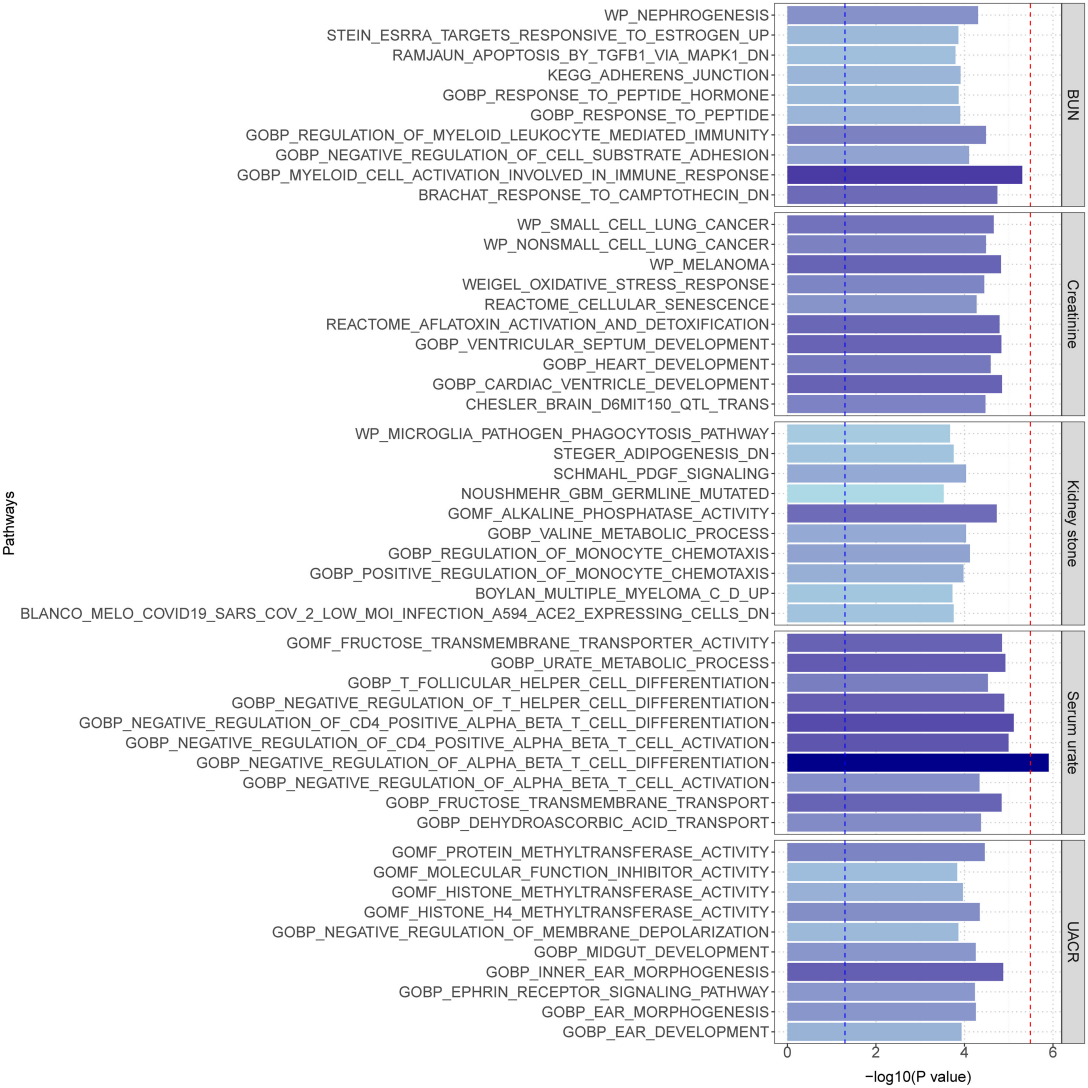
MAGMA pathway enrichment (top 10). The blue line represents a significance level of 0.05, and the red line indicates results that remain significant after multiple corrections (P< 0.05/15485 = 3.23E-6).

We further conducted MAGMA gene set enrichment analysis on the pleiotropic results using Gene Ontology terms, presenting the top 10 significantly enriched gene sets in **Figure 4** and **Supplementary Table S4**. Functional enrichment analysis revealed that the most significant set for BUN was “GOBP_MYELOID_CELL_ACTIVATION_INVOLVED_IN_IMMUNE_RESPONSE” (p=4.95×10^-6^), while for creatinine, it was “GOBP_CARDIAC_VENTRICLE_DEVELOPMENT” (p = 1.42×10^-5^). For serum urate, the most significant was “GOBP_NEGATIVE_REGULATION_OF_ALPHA_BETA_T_CELL_DIFFERENTIATION” (p=1.25×10^-6^), and for UACR, the most significant was “GOBP_INNER_EAR_MORPHOGENESIS” (p=1.33×10^-5^). Finally, for kidney stone, the most significant enriched gene set was “GOMF_ALKALINE_PHOSPHATASE_ACTIVITY” (p=1.87×10^-5^). Tissue-specific MAGMA analysis revealed significant enrichment of maternal effects in the pleiotropy of the 5 pairs of results in kidney tissue. Additionally, reports of enrichment were observed in other tissues such as bladder, liver, prostate, and thyroid, among others (**Supplementary Figure S4**). Notably, the MAGMA gene set and tissue-specific analyses in this section were conducted using the complete distribution of SNP p-values.

We leveraged positional information of lead SNPs to identify genes associated with pleiotropic risk loci across the five significantly correlated pairs. A total of 28 genes were mapped to these loci, and details are provided in the last column of **Table 1** (also available in **Supplementary Table S5**). We further employed MAGMA gene-set analysis to identify pleiotropic genes with genome-wide significance (**Supplementary Table S6**, **Supplementary Figure S5**). This analysis identified 33 pleiotropic genes (listed in **Supplementary Table S6**, **Supplementary Figure S5**). The QQ plot for the MAGMA gene-test is presented in **Supplementary Figure S6**. Additionally, we used eQTL information from whole blood tissue to explore genes potentially regulated by these pleiotropic risk loci. Specific details are provided in **Supplementary Table S5**.

**Figure 5:**
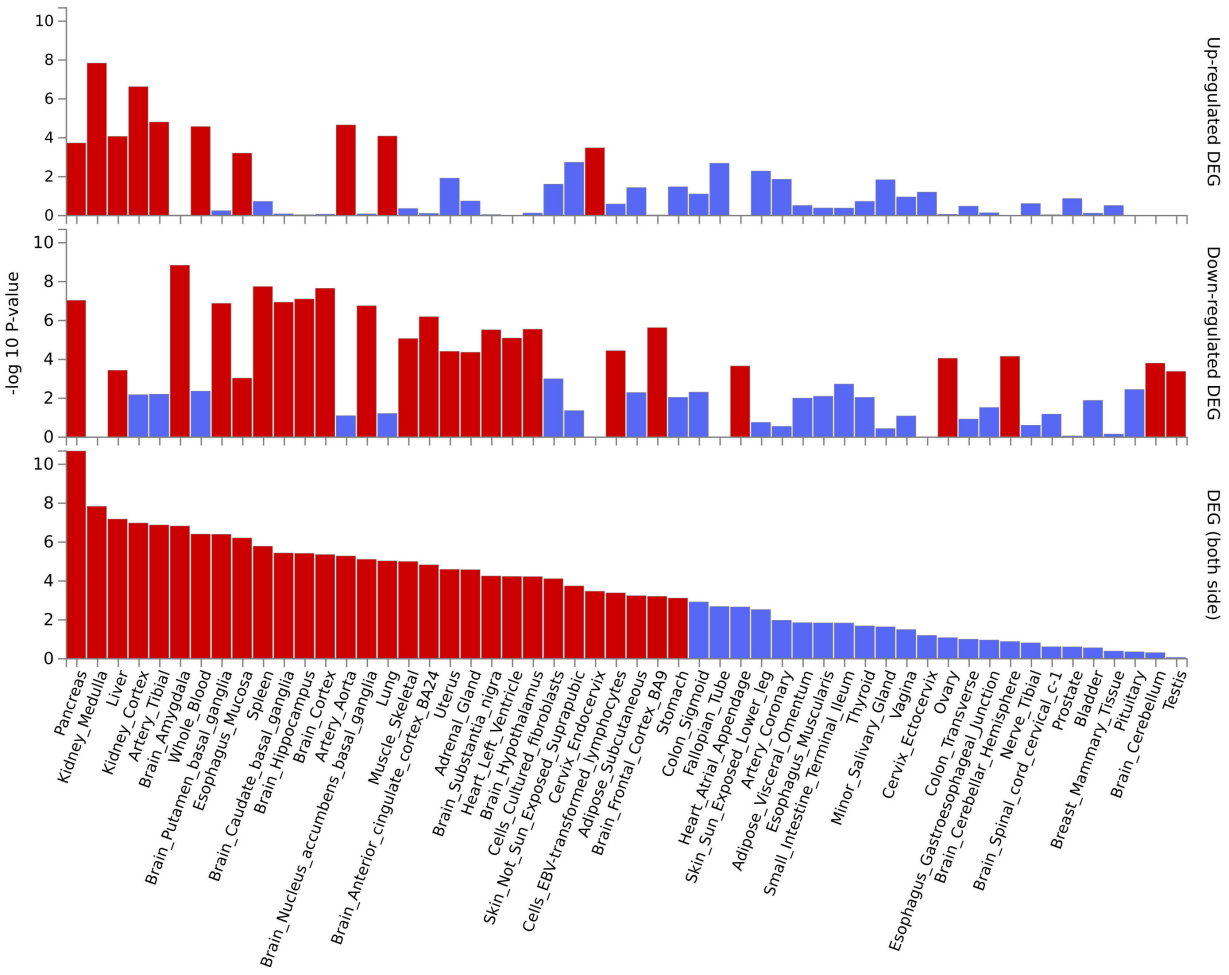
The enrichment of pleiotropic genes (based on MAGMA gene testing) across different tissues.

Several pleiotropic genes displayed significant differential expression in tissues relevant to our study, such as the kidney and pancreas (**Supplementary Figure S7**, **Supplementary Table S7**). This finding is further supported by tissue-specific enrichment analysis, which revealed an enrichment of these positionally mapped genes in pancreas and kidney tissues (**Figure 5**, **Supplementary Table S7**). Pathway enrichment analysis, based on MAGMA gene testing, is illustrated in **Supplementary Figure S8**. The most significant enrichment was observed in “8p23.1 copy number variation syndrome”. Cell-type enrichment analysis results are displayed in **Supplementary Figure S9**, with the most notable enrichment found in “Aizarani liver C4 Epcam pos bile duct cells1”. Finally, the protein-protein interaction (PPI) network analysis in **Supplementary Figure S10** revealed a network of six genes that interact with each other.

**Figure 6:**
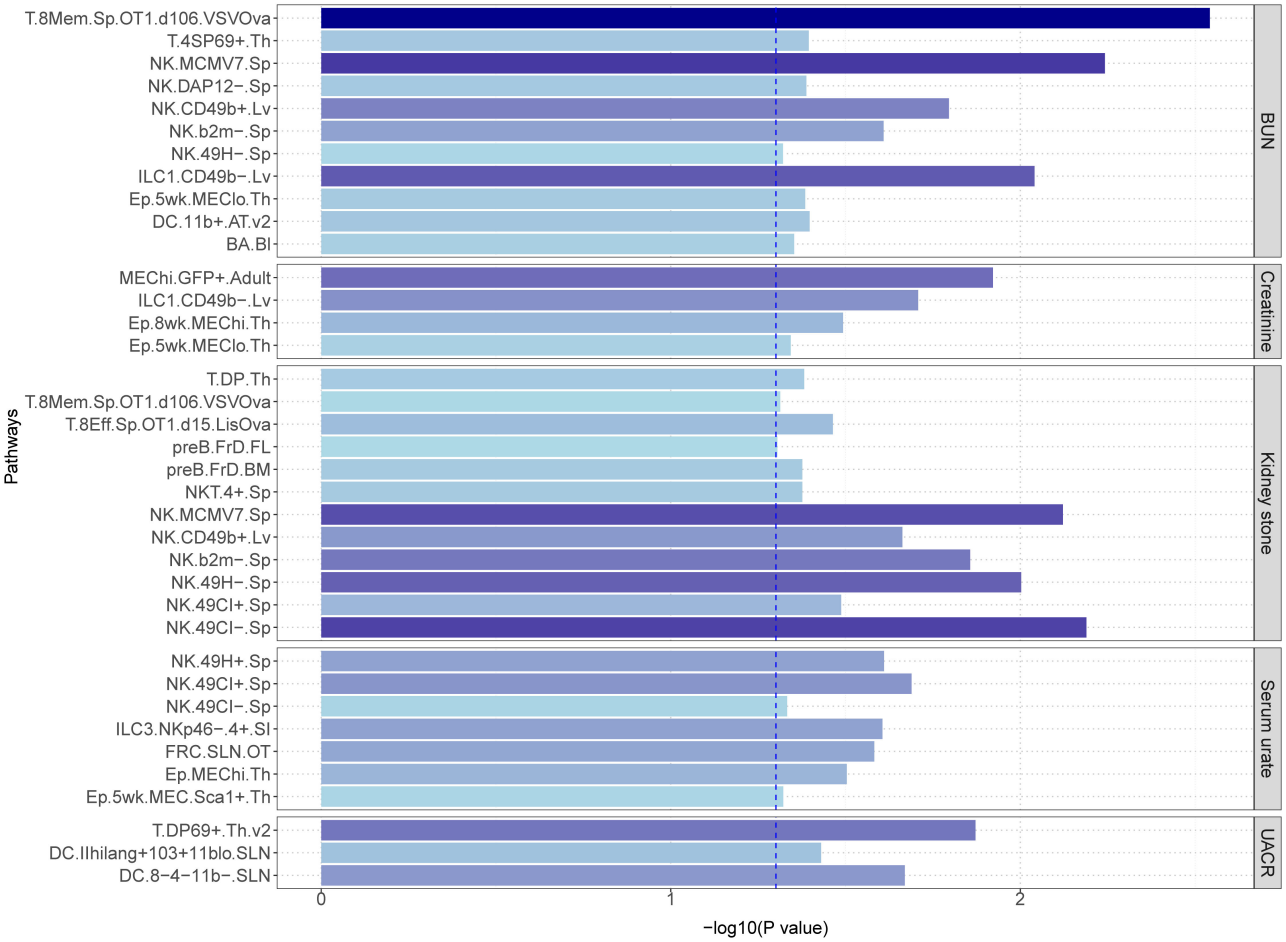
The LDSC cell-type-specific heritability enrichment analysis.

To further explore the immunological underpinnings of these associations, we conducted LDSC immune cell-type-specific enrichment analyses for sepsis and the kidney-related traits (**Figure 6**). We investigated a total of 37 immune cell types in relation to these phenotypes. Among the traits, BUN exhibited associations with 11 immune cell types, with “T.8Mem.Sp.OT1.d106.VSVOva” showing the strongest enrichment (p = 2.87×10^-3^). Similarly, significant immune cell enrichments were observed for Creatinine (4 identified cells, most significant: “MEChi.GFP+.Adult”, p = 1.20×10^-2^), Serum urate (7 identified cells, most significant: “NK.49CI+.Sp “, p = 2.05×10^-2^), UACR (3 identified cells, most significant: “T.DP69+.Th.v2”, p = 1.34×10^-2^), and Kidney stone (12 identified cells, most significant: “NK.49CI-.Sp”, p = 6.47×10^-3^). Interestingly, the analysis also revealed shared immune cell enrichments between certain traits. For example, “T.8Mem.Sp.OT1.d106.VSVOva, NK.MCMV7.Sp, NK.CD49b+.Lv, NK.b2m-.Sp” cells were enriched for both BUN and Kidney stone, while “NK.49CI+.Sp” and “NK.49CI-.Sp” were enriched for both Serum urate and Kidney stone. Additionally, “ILC1.CD49b-.Lv” and “Ep.5wk.MEClo.Th” cells showed enrichment for both BUN and Creatinine. Full details of all identified immune cell enrichments can be found in **Supplementary Table S9**.

### 3.3 MR analysis

We employed the two-sample Mendelian randomization method to investigate the causal relationship between the kidney-related diseases and sepsis. However, the IVW analysis did not identify a statistically significant causal association between them (**Supplementary Table S10**). Sensitivity analyses yielded results consistent with the main findings, showing no evidence of directional pleiotropy or heterogeneity (**Supplementary Table S11**).

## 4 Discussion

This study investigated the complex genetic relationship between kidney diseases and sepsis. We employed two main approaches: first, LDSC analysis to evaluate their genetic correlation, and second, a pleiotropy approach to identify shared genetic loci and genes potentially influencing both conditions. We then assessed causal associations through a two-sample bidirectional MR method. Our analyses revealed significant genetic correlations between BUN, creatinine, urine albumin, UACR, kidney stones, and sepsis. We identified 24 risk loci across five pairs of significantly correlated traits, including four loci shared by multiple traits. Further integrated analysis using MAGMA gene-test and eQTL data identified *PPP2R3A*, *VAMP8*, *DOCK7*, and *HIBADH* as shared risk genes. Genetic enrichment analysis revealed 50 potentially relevant pathways and 37 immune cell types associated with both diseases. These findings suggest a shared genetic architecture and potentially overlapping pathogenic mechanisms between kidney diseases and sepsis. This deeper understanding of pleiotropy in sepsis paves the way for future research on disease prevention.

Genetic correlation analysis revealed a significant positive genetic correlation between BUN, creatinine, UACR, serum urate, kidney stones, and sepsis. Previous studies have also reported associations among these factors, and we have summarized the findings of these studies in **Supplementary Table S1** of the **Supplementary Files**. Our findings align with and further validate the results of these aforementioned studies. While existing reports suggest relationships between eGFR, IgA nephropathy, kidney neoplasm, diabetic nephropathy, and sepsis (26–29), our study did not identify significant connections among them. Several factors may explain these null findings: sample size limitations, population structure complexities, disease heterogeneity, and potential confounding or reverse causation inherent to observational studies could all play a role. For instance, diabetic nephropathy often results from diabetes, and the genetic correlation between diabetes and sepsis might be insignificant. Additionally, our kidney neoplasm data included cases of metastasized tumors, potentially introducing inaccuracies. The lack of association with IgA nephropathy could be due to a true lack of correlation or its complex etiology. Interestingly, while eGFR showed no significant genetic correlation with sepsis, serum creatinine levels did. This might be because eGFR is also influenced by factors like age and weight, impacting the results. Therefore, these non-significant relationships were not elaborated in the following discussion. Although MR analysis did not establish causality between kidney diseases and sepsis, our genetic correlation and pleiotropy analyses revealed a shared genetic architecture. This suggests that multiple risk loci contribute to both conditions, indicating a potential pleiotropic effect. Individuals with specific mutations for kidney diseases may also have an increased risk of sepsis, and vice versa. However, this shared genetic architecture does not imply that kidney disease and sepsis are inevitable co-occurrences. Clinicians should remain vigilant for potential sepsis in patients with persistently elevated serum creatinine, BUN, UACR or serum urate, as well as those with kidney stones. Prompt antibiotic use to prevent infections is advisable, rather than relying on advanced antibiotics after sepsis develops, which can contribute to bacterial resistance.

PLACO pleiotropy analysis of the five significant trait pairs identified 24 pleiotropic loci and 28 potentially associated genes. Notably, a shared risk locus (11p14.1) and common genes (*RP5–1024C24.1*, *DCDC1*) were found in both BUN and creatinine. While not previously linked to sepsis, this locus has been associated with sex hormone secretion (30). The gene *RP5–1024C24.1* lacks current research, while DCDC1 has been implicated in esophageal cancer (31). Similarly, BUN and UACR shared a risk locus (4q21.1) and the gene *SHROOM3*. This locus has been previously linked to essential tremor (32), while *SHROOM3* is known to play a role in kidney function and chronic kidney disease (33). Our findings suggest a potential novel genetic link between *SHROOM3* and sepsis. BUN and serum urate shared another risk locus (12q13.3) and the gene R3HDM2. This previously unreported locus for BUN, serum urate, and sepsis is a novel finding for nodular disease (34). *R3HDM2*, linked to uric acid transport (35), aligns with our investigation and strengthens the potential genetic connection between BUN and sepsis. Creatinine and UACR shared a common risk locus (13q14.3) without identified nearby genes. While currently associated with leukemia (36), there is no existing evidence linking 13q14.3 to creatinine, UACR, or sepsis. These newly discovered loci and genes hold promise as future therapeutic targets for sepsis, warranting further investigation.

MAGMA gene-testing, whole blood tissue eQTL information, and analysis of nearby genes all converged on four genes: *PPP2R3A*, *VAMP8*, *DOCK7*, and *HIBADH*. A mouse model study suggested *PPP2R3A*’s role in initiating lung inflammation (37). While *VAMP8* has been linked to inflammation in periodontal disease (38). DOCK7 is associated with serum lipid level (39), which are known to play a role in both sepsis and kidney health (40). Finally, *HIBADH*, previously connected to kidney stones (41), has been documented to be involved in valine metabolism disorders (42),a pathway recently implicated in sepsis (43). The established roles of these genes in inflammation, lipid metabolism, and metabolic disorders, alongside our findings in sepsis and kidney diseases, suggest a potential interplay in the pathogenesis of these conditions. However, further research is required to elucidate these potential connections. While tissue expression analysis revealed significantly higher levels of certain pleiotropic genes, including *RAP1GAP*, *PBXIP1*, *VAMP8*, *SUMO1*, *SPP1*, and *SCARB2*, in kidney, pancreas, and liver tissues, tissue-specific enrichment analysis further confirmed this enrichment for these positionally-mapped genes. Notably, *VAMP8* emerged as a common pathogenic gene across all three methods employed. This gene exhibits high expression in the kidneys, but its precise physiological and molecular roles remain unclear (44). Interestingly, research suggests its interaction with apolipoproteins, potentially contributing to chronic kidney disease (45). Our study additionally identified its association with the pancreas, which aligns with previous reports highlighting its role in pancreatitis (46). This potentially explains the frequent clinical observation of pancreatitis progressing to sepsis and subsequent renal dysfunction. Moreover, genes like *RAP1GAP*, expressed in multiple tissues, have been linked to glomerular damage (47). Similarly, *PBXIP1* is associated with renal fibrosis and chronic kidney disease (48), while *SPP1* is related to damage in renal tubules (49). These co-expressed genes, along with *VAMP8*, suggest an intrinsic link between the kidneys and other organs, potentially explaining the multi-organ dysfunction observed in sepsis and the need for diverse clinical indicators for diagnosis. However, further research is crucial to fully elucidate this intricate relationship.

Several gene sets were enriched across the 5 pairs of results. T cells are categorized into αβT cells and γδT cells based on the expression of their T-cell receptors. γδT cells, known to be associated with sepsis (50), have been implicated in this process. While αβT cells are more prevalent in the kidney and linked to acute kidney injury (51), their specific role in sepsis remains unclear. Our findings suggest that serum urate, possibly through its influence on αβT cells, could play a role in sepsis development. Myeloid cells are another enriched pathway identified in our analysis. Established research confirms their involvement in sepsis (52). Consequently, elevated blood urea nitrogen levels may modulate myeloid cell function, potentially influencing susceptibility to sepsis. Interestingly, the link between creatinine and ventricular function emerged from our analysis. Studies in animal models with reduced kidney function (5/6 nephrectomy) observed left ventricular hypertrophy and progressive creatinine elevation (53). Similarly, septic cardiomyopathy with altered ventricular morphology is a common observation in sepsis patients (54). Our findings suggest a potential underlying mechanism where increased creatinine levels might impact ventricular development, influencing susceptibility to sepsis. This hypothesis warrants further investigation. Intriguingly, MAGMA analysis identified the enrichment of ion channel and transport protein pathways. These proteins are abundantly expressed in both the inner ear and the kidneys (55). A study involving Alport mice with glomerular disease and hearing loss (56) demonstrated that dual inhibition of endothelin and angiotensin receptors improved kidney and inner ear lesions. This suggests a link between the kidneys and the inner ear. In a mouse model of sepsis, observations indicated that sepsis can affect the morphology and function of the inner ear. We speculate that there might be a link connection between UACR, inner ear morphology, and sepsis. In a retrospective analysis, alkaline phosphatase was identified as a predictive factor for stone formation (57). Interestingly, research has explored its potential role in treating sepsis-related acute kidney injury (58), suggesting its potential as a novel therapeutic target for inflammation in sepsis-related kidney complications (59). Therefore, our study suggests that kidney stones might alter alkaline phosphatase, thereby influencing sepsis. At present, we have only discussed the most significant pathways in the pathway analysis among the five pairs of results. No overlapping pathways were found among the five pairs of results. Nevertheless, all enriched pathways hold promise as potential therapeutic targets for sepsis, warranting further investigation. While our pathway analysis focused on kidney-related genes, enrichment signals were also observed in other tissues such as bladder, liver, prostate, and thyroid. This suggests a broader influence beyond the kidneys, potentially involving a complex interplay between multiple organs in sepsis. The close association of BUN, creatinine, serum urate, UACR, and kidney stones with renal function strengthens the link between the kidneys and sepsis. Our findings suggest a potential genetic connection, where sepsis can trigger changes in these well-established kidney function indicators. This aligns with common observations in retrospective studies, where alterations in these markers frequently accompany sepsis events (6, 60–62). Furthermore, diseases that frequently alter these indicators may also predispose individuals to sepsis. Clinicians should therefore maintain a heightened awareness for potential sepsis development when encountering changes in these established markers and implement timely preventive measures. The insights gleaned from our results hold promise for guiding the development of effective treatments targeting these identified associations. Pathway enrichment analysis of pleiotropic genes identified by MAGMA gene testing revealed a significant association with “8p23.1 copy number variation syndrome”. Similarly, cell-type enrichment analysis highlighted “Aizarani liver C4 Epcam pos bile duct cells1”. However, no research has explored their specific connection to sepsis. While PPI network analysis identified six interacting genes, only HFE has established links to both chronic kidney disease (63) and sepsis (64). The absence of existing literature on the remaining genes should not preclude the inference of a potential genetic link between the kidney and sepsis based on their interactive network. Further research is thus necessary to elucidate the specific roles of these genes, pathways, and cell types in the context of sepsis.

The most significant cell type, “T.8Mem.Sp.OT1.d106.VSVOva”, observed in BUN and sepsis, also showed significance in kidney stone. This cell type is closely linked to CD8 T cells in the spleen. Intriguingly, CD8 T cells have been independently implicated in sepsis (65), BUN (66), and kidney stones (67), suggesting a potentially crucial role for these cells in the interplay between these conditions. Creatinine and sepsis exhibited the most significant enrichment for the immune cell type, “MEChi.GFP+.Adult”, while “ILC1.CD49b-.Lv” was co-expressed in BUN and creatinine. Current research on these two cell types is limited, and their specific relevance to sepsis remains to be elucidated. Analysis of serum urate and sepsis revealed “NK.49CI+.Sp” as the most significant cell type, which was also enriched in kidney stones. Similarly, kidney stones exhibited the most significant enrichment for “NK.49CI-.Sp”, a cell type co-expressed with serum urate. Additionally, other NK cells identified in kidney stones also showed expression in BUN. These findings collectively suggest a potential role for NK cells in the interplay between these conditions. Supporting this notion, previous studies have established the importance of NK cells in sepsis. David et al. demonstrated that NK cell count can predict mortality rates in severe sepsis (68). Furthermore, single-cell RNA sequencing studies on sepsis highlight the immunomodulatory role of NK cells (69). A connection between uric acid and NK cells has also been documented. A single-cell sequencing study of gout patients identified NK cells as potential contributors to novel therapeutic targets (70), and a mouse model demonstrated that NK cells can ameliorate gout inflammation (71). However, research on the correlation between kidney stones, BUN, and NK cells is sparse. A study on urinary tract stones with infection noted changes in NK cells, but the specific mechanisms were not explored (72). Another study investigating risk factors for death in COVID-19 patients found that elevated urea nitrogen and NK cells correlated with a poor prognosis (73). Finally, the immune cell type “T.DP69+.Th.v2” emerged as the most significant finding in the UACR and sepsis analysis. Currently, no relevant research on this specific cell type is available.

Our study has several limitations that should be acknowledged. First, the participants were predominantly of European descent. Since sepsis and kidney issues are global concerns, generalizability of these findings to other populations is crucial and requires further investigation. Second, due to limitations in acquiring individual-level data and the lack of large, well-characterized cohorts, our study relied heavily on publicly available GWAS databases. This limited the ability to perform subgroup or stratified analyses. Third, the MR analysis did not establish a causal relationship between kidney diseases and sepsis, suggesting a potential comorbid association rather than a direct causal effect. Fourth, some of the newly identified gene loci, pathways, and immune cells lack prior mention in existing literature. In the future, we aim to further explore and validate our findings through additional studies. Validation of these findings necessitates additional, well-designed experiments or clinical research. Despite these limitations, this study offers the best available evidence to date on genetic correlations between kidney diseases and sepsis.

## 5 Conclusion

By integrating comprehensive GWAS data and applying diverse methodologies, our study identified novel pleiotropic loci, shared pathogenic genes, enriched pathways, and immune cells. This comprehensive analysis sheds light on the complex interplay between kidney disease and sepsis, potentially revealing a shared underlying etiology. These findings offer valuable insights for early intervention and treatment strategies, paving the way for further research in this critical area.

## Data availability statement

The datasets presented in this study can be found in online repositories. The names of the repository/repositories and accession number(s) can be found in the article/**Supplementary Material**.

## Ethics statement

In our study, we utilized large-scale GWAS datasets rather than individual-level data. The studies included in these consortia obtained approval from local research ethics committees and institutional review boards, with all participants providing written informed consent.

## Author contributions

TZ: Conceptualization, Writing – original draft, Writing – review & editing. YC: Data curation, Formal analysis, Writing – original draft. SJ: Data curation, Formal analysis, Writing – review & editing. LJ: Data curation, Formal analysis, Writing – review & editing. LS: Data curation, Formal analysis, Writing – review & editing. LH: Visualization, Writing – review & editing. YL: Visualization, Writing – review & editing. JY: Writing – original draft. ML: Conceptualization, Writing – original draft, Writing – review & editing.
