# Supplementary material for: Shared genetic correlations between kidney diseases and sepsis
Source: Front Endocrinol (Lausanne). 2024 Jul 17;15:1396041. doi: 10.3389/fendo.2024.1396041 (PMC11288879; doi:10.3389/fendo.2024.1396041)
Supplement: Supplementary file 2 [file Table_2.docx]

Table 1 The five pairs of results literature review

| **Trait pairs** | **Pertinent description** | **Years References** | |
| --- | --- | --- | --- |
|  | The levels of blood urea nitrogen were found to be closely associated with the occurrence and severity of neonatal sepsis.  The correlation between blood urea nitrogen levels and 30-day mortality in patients with sepsis.  Blood urea nitrogen is an independent marker of mortality in sepsis.  Blood urea nitrogen and creatinine could predict the severity and outcomes of abdominal sepsis in rats.  Blood urea nitrogen is a predictor of sepsis-associated liver injury.  The renal injury scores, serum creatinine, and blood urea nitrogen, were higher in septic mice than in non-septic mice.  The blood urea nitrogen-to-serum albumin ratio is significantly associated with in-hospital mortality in critically ill septic patients. | | 2022 Xiaojuan et al (1)  2021 Xu et al (2)  2023 Martin et al (3)  2012 Min et al (4)  2022 Tonghui et al (5)  2023 Yuting et al (6)  2022 Shaoyan et al (7) |
| **BUN&Sepsis** | Blood urea nitrogen was found to be significantly elevated only in the group of burn patients with positive blood cultures among those with sepsis, as compared to those with negative blood cultures. | | 2023 Jaechul et al (8) |
|  | IL-6, blood urea nitrogen and mean arterial pressure were independent risk factors for sepsis diagnosis.  The cecal ligation and puncture model in septic mice increases levels of blood urea nitrogen, serum creatinine, and pro-inflammatory cytokines in the serum.  Elevated levels of blood urea nitrogen and creatinine were observed in the septic mouse model. | | 2022 Baozhong (9)  2022 Li et al (10)  2022 Jin-Ling et al (11) |
|  | Blood urea nitrogen is used to predict the prognosis of sepsis in patients with pyogenic liver abscess. | | 2023 Ji et al (12) |
|  | The 28-day mortality rate in sepsis was 48%, correlating with admission lactate, blood urea nitrogen, and creatinine levels, but independent of cardiovascular status.  Higher blood urea nitrogen is a risk factor for sepsis during severe malaria in adults.  Early septic mice were divided into groups, and compared to mice predicted to survive, those predicted to die had blood urea nitrogen levels nearly five times higher within 24 hours. | | 2019 Fabio et al (13)  2022 Tsi et al (14)  2014 Florin et al (15) |
|  | Elevated serum creatinine is an independent factor associated with a poorer prognosis in sepsis. | | 2022 Diomidis et al (16) |
|  | Elevated serum creatinine is an independent factor associated with a poorer Elevated creatinine >1.5 mg/dl is an independent predictive factor for sepsis in patients with SARS-CoV-2 infection. | | 2021 Mohammad et al (17) |
|  | Elevated creatinine is significantly correlated with a higher mortality rate in neonatal sepsis patients. | | 2022 Dariela et al (18) |
| **Creatinine&Sepsis** | Elevated creatinine and blood urea nitrogen are risk factors associated with an increased risk of sepsis in elderly patients after emergency surgery. | | 2022 Xiaorong et al (19) |
|  | Persistent elevation of creatinine is associated with radiation pneumonitis and sepsis. | | 2022 Yufei et al (20) |
|  | Elevated creatinine in patients with severe sepsis or septic shock. | | 2015 Daniel et al (21) |
|  | Higher creatinine levels (median: 2.300 mg/dl) in patients with sepsis-related ARDS in the ICU are associated with in-hospital mortality.  The fluctuation of creatinine is an important characteristic in determining the onset of sepsis in patients.  The survival rate of septic mice in the cecal ligation and puncture model is low, with increased levels of blood urea nitrogen and creatinine.  The risk of doubling serum creatinine in the sepsis group is higher than in the non-sepsis group.  Elevated serum creatinine (≥1.5) was associated with post-cardiac surgery sepsis. | | 2022 Yu et al (22)  2023 Ali et al (23)  2018 Chaoyang et al (24)  2022 Shuo-Ming et al (25)  2018 Sergey et al (26) |
| **UACR&Sepsis** | The infection-related mortality rate is higher when the urinary albumin-to-creatinine ratio levels are in the range of 30-299 and ≥300 mg/g. | | 2012 Henry et al (27) |
|  | After sepsis, acute kidney injury is more likely to occur with elevated urinary albumin-to-creatinine ratio. | | 2014 T Clark et al (28) |
|  | Febuxostat users and allopurinol users are associated with a reduced risk of sepsis/infection. | | 2022 Huang e al (29) |
|  | The uric acid levels in the sepsis group are significantly higher than those in the control group. | | 2001 A et al (30) |
| **UA&Sepsis** | Elevated serum uric acid levels in sepsis patients upon admission are positively correlated with adverse outcomes and prolonged hospitalization.  In sepsis patients in the intensive care unit, hyperuricemia is significantly associated with an increased risk of 90-day all-cause mortality and the incidence of acute kidney injury.  In sepsis patients, elevated serum uric acid levels may be associated with systemic inflammation.  Uric acid crystals can promote inflammation by activating the NLRP3 inflammasome, and soluble uric acid can induce the production of CCL2 to drive monocyte aggregation.  Hyperuricemia may serve as an early marker for predicting mortality and incidence in sepsis patients.  Uric acid levels may only serve as an additional tool to support the diagnosis of neonatal sepsis.  Elevated serum uric acid levels serve as an early indicator of the severity of sepsis. | | 2022 Jayprakash et al (31)  2022 Shizhen et al (32)  2021 Emre et al (33)  2020 Xu et al (34)  2022 [Sreekanth](https://pubmed.ncbi.nlm.nih.gov/?term=Sreekanth&cauthor_id=35443484) et al (35)  2014 Banu et al(36)  2015 Sana et al (37) |
| **Kidney stone&Sepsis** | An untreated staghorn calculus can lead to kidney damage over time, resulting in a decline in kidney function and potentially causing life-threatening sepsis.  More than half of the 42 individuals who died from stone disease succumbed to sepsis.  Patients with complex stones and positive urine cultures are significantly associated with an increased risk of postoperative sepsis.  A stone diameter greater than 6 cm is more likely to lead to urosepsis after percutaneous nephrolithotomy.  Struvite stone patients face a significantly heightened risk of stone recurrence and sepsis after surgery.  The existence of kidney stones in critically ill older adults is often linked to urosepsis and septic shock. | | 2020 Arvind et al (38)  2022 Radhika et al (39)  2020 Jun et al (40)  2023 Xinfeng et al (41)  2022 Xiaomin et al (42)  2021 Gurhan et al (43) |

Figure S1.  The QQ plots for the 5 pairs of pleiotropy results.


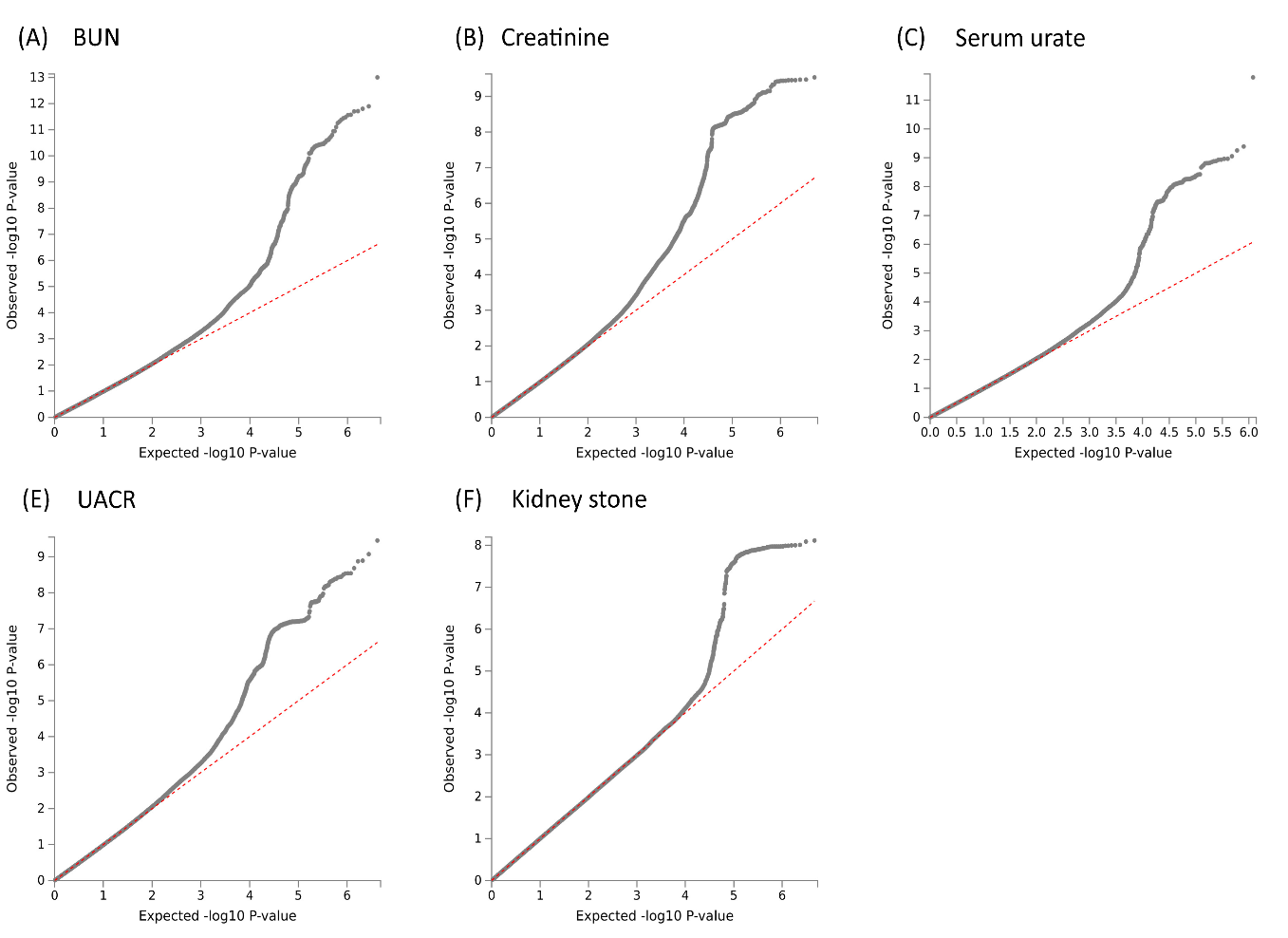


Figure S2. The basic information summary for each genomic risk locus (from left to right, representing the size of the risk locus, the number of SNPs, the number of mapped genes, and the number of genes within the locus).


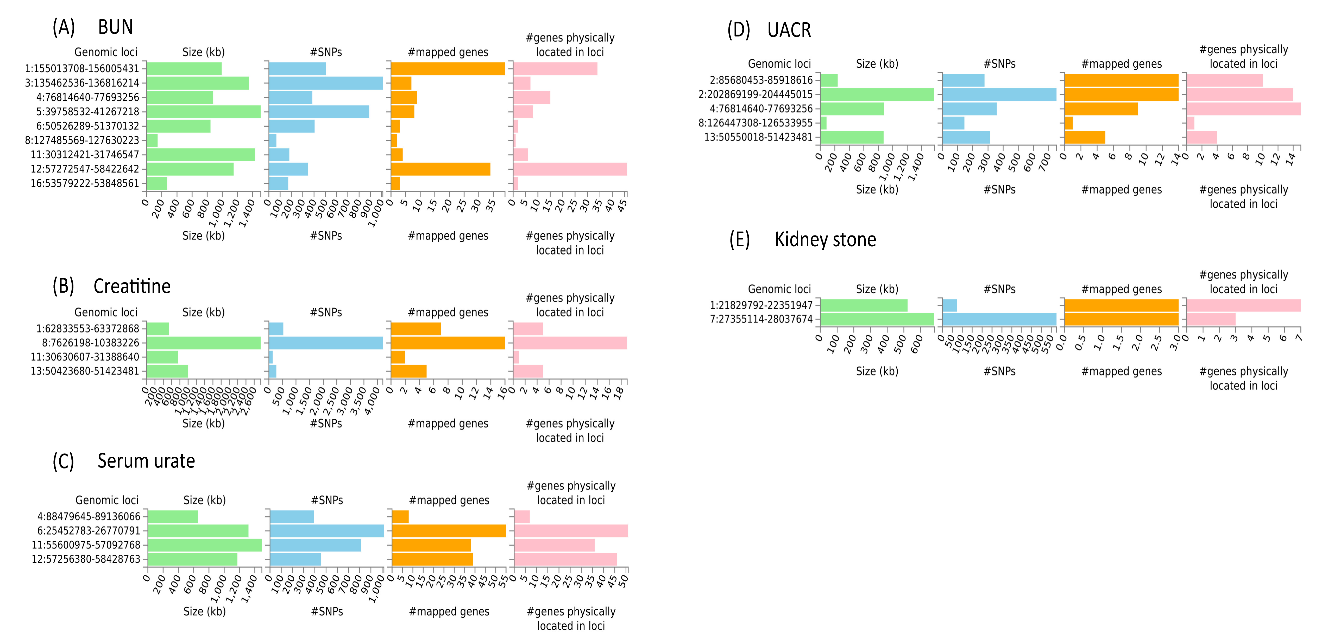


Figure S3. The impact of pleiotropic SNPs on gene function.


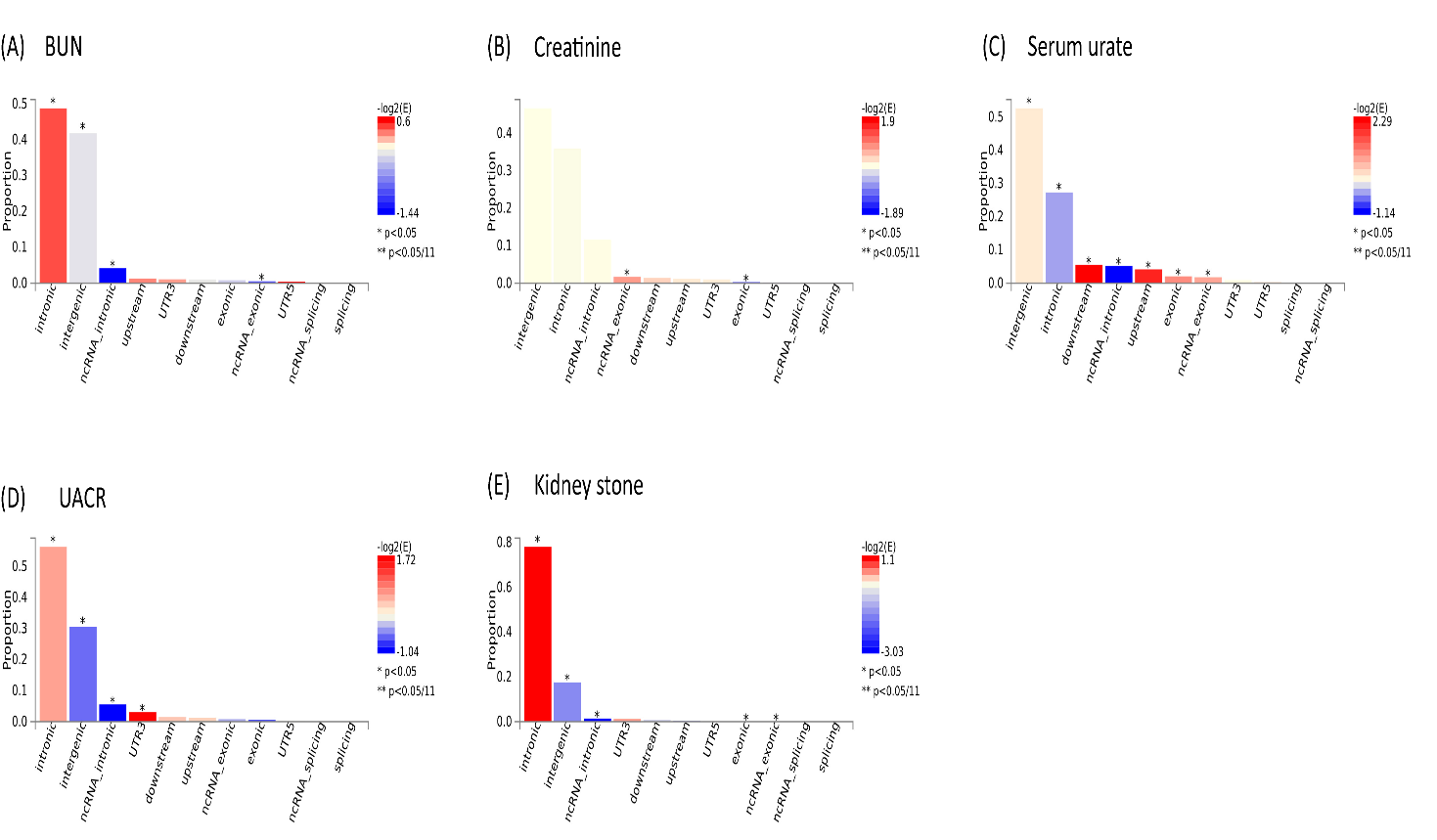


Figure S4. MAGMA analysis reveals the pleiotropy enrichment of 5 pairs of results in different tissues


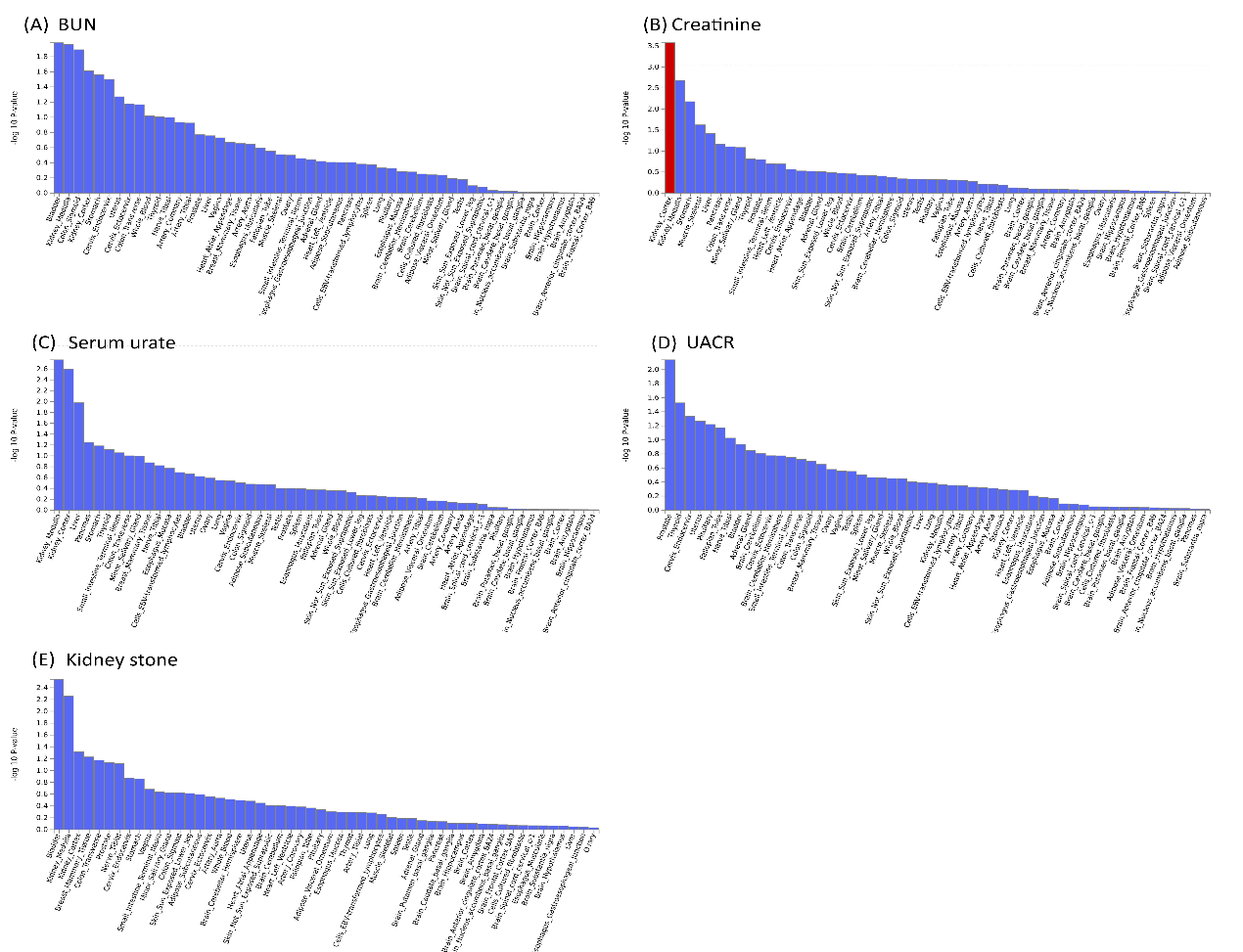


Figure S5. Manhattan plots of pleiotropic genes for 5 pairs of results (based on MAGMA gene testing)


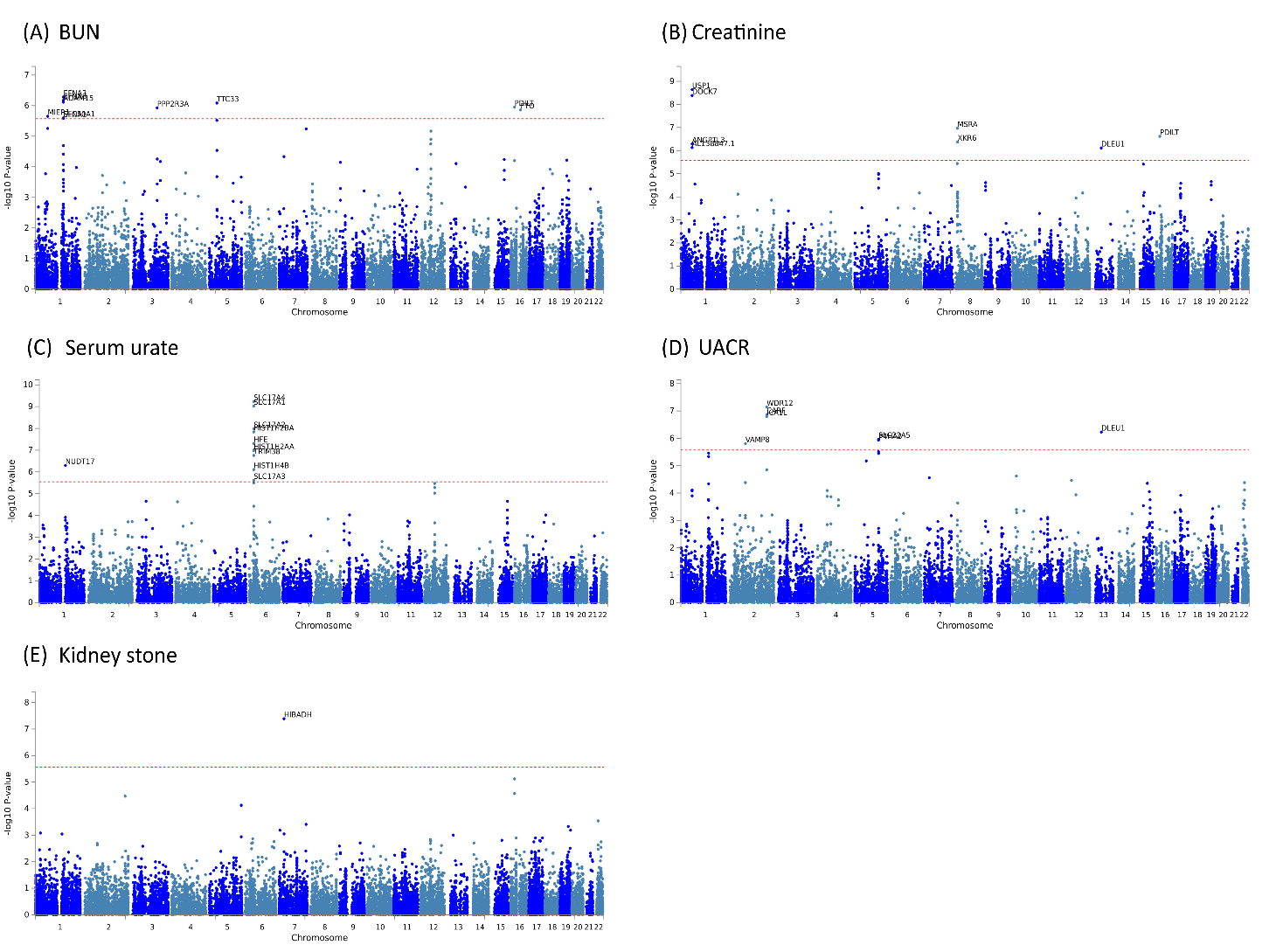


Figure S6. QQ plots of pleiotropic genes for 5 pairs of results (based on MAGMA gene testing


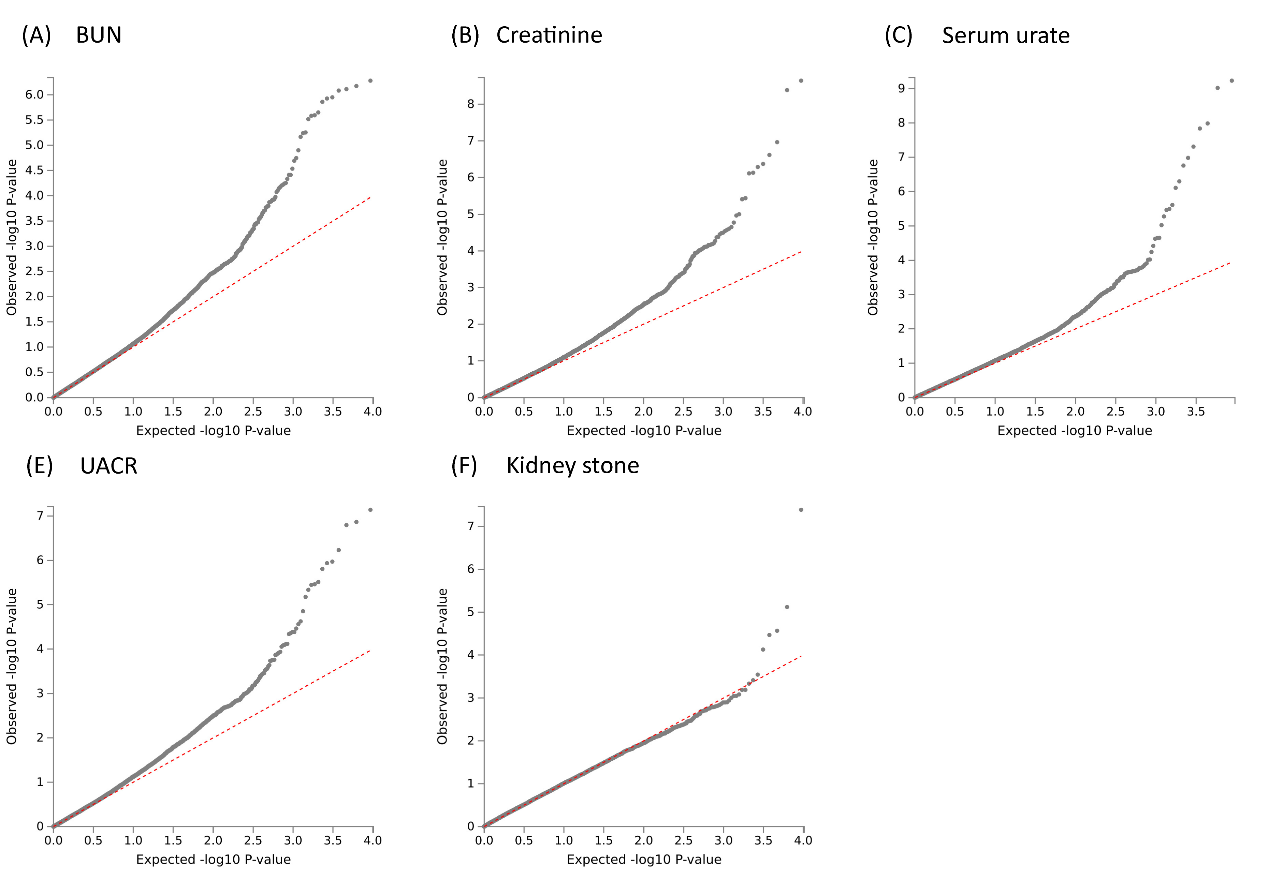


Figure S7. The expression profiles of pleiotropic genes, based on MAGMA gene testing, across various tissues.


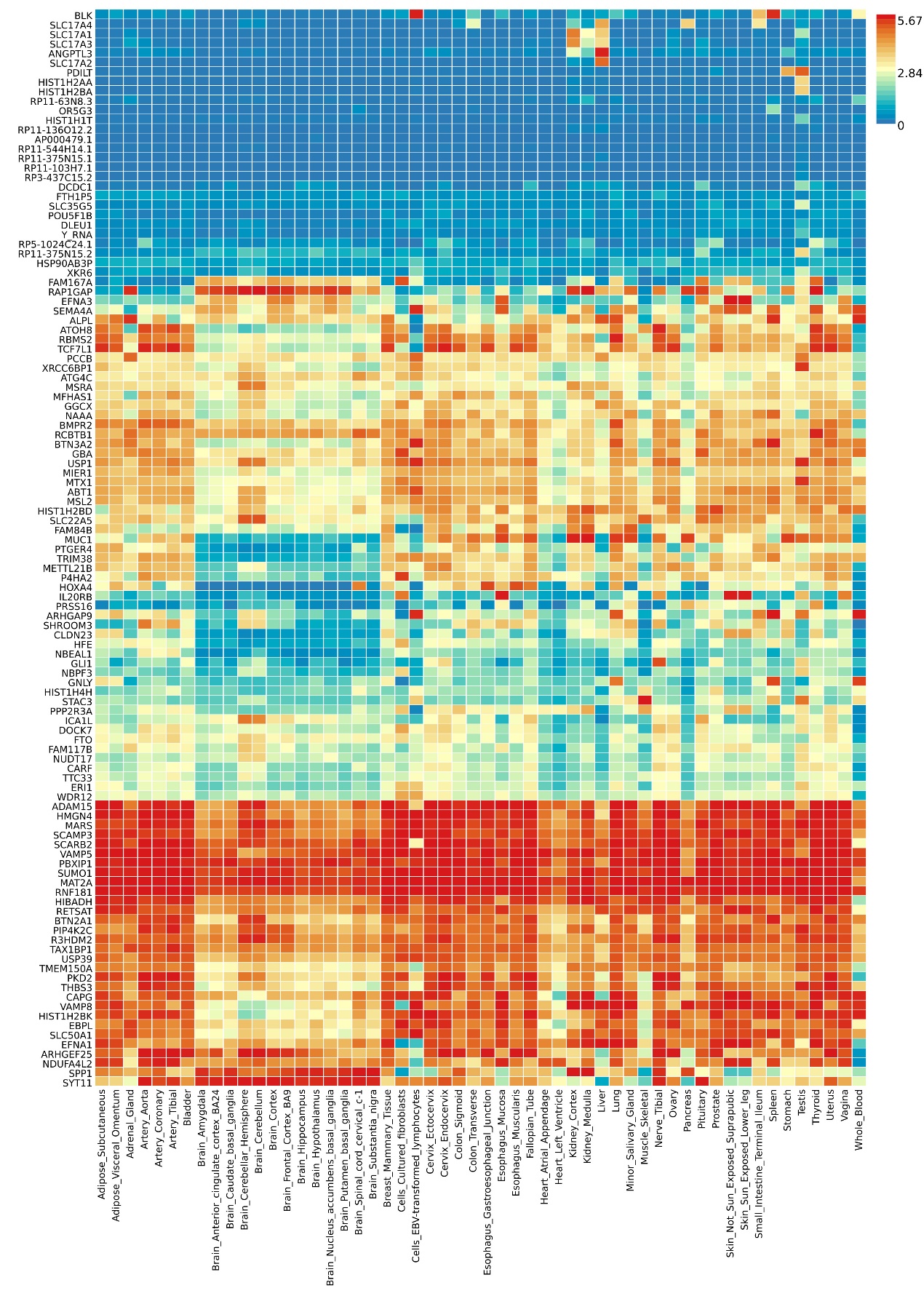


Figure S8. Pathway enrichment analysis (KEGG, Wiki, GO) of pleiotropic genes based on MAGMA gene testing
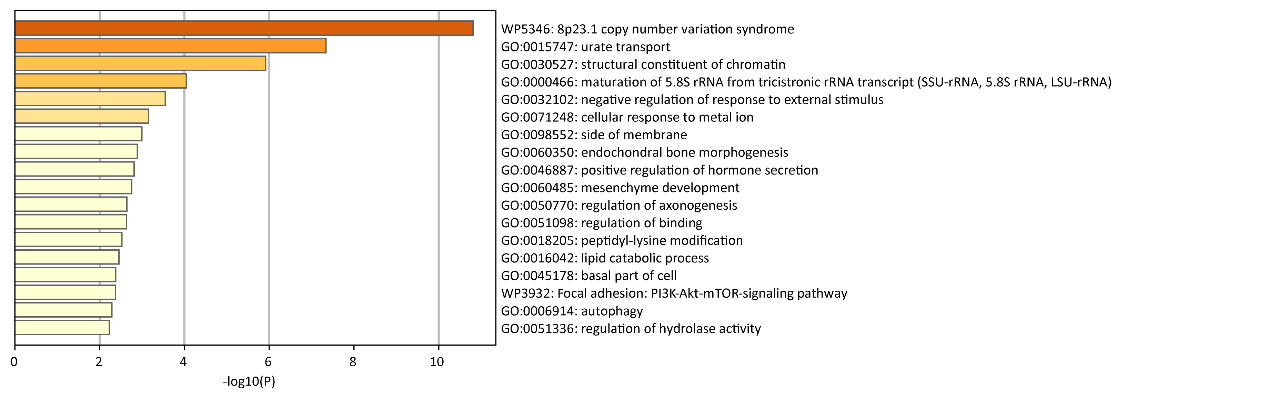


Figure S9. Cell-type enrichment analysis of pleiotropic genes based on MAGMA gene testing.
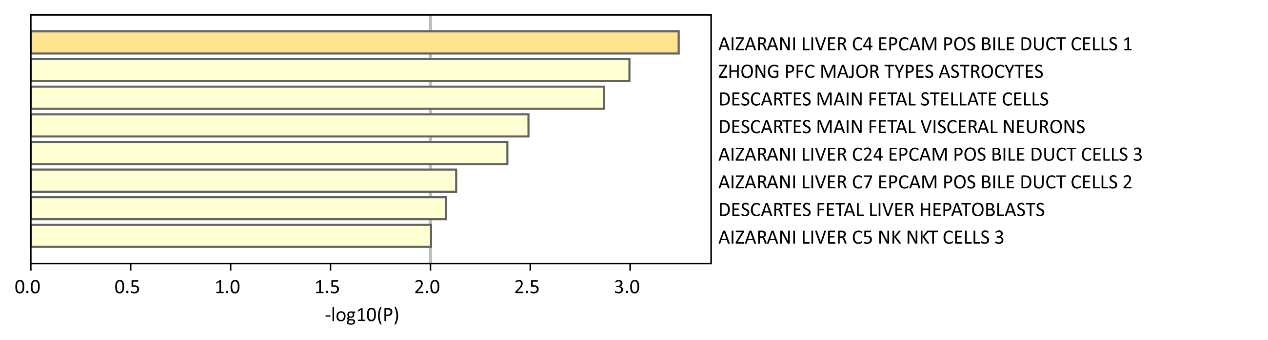


Figure S10. Protein-Protein Interaction network analysis of pleiotropic genes


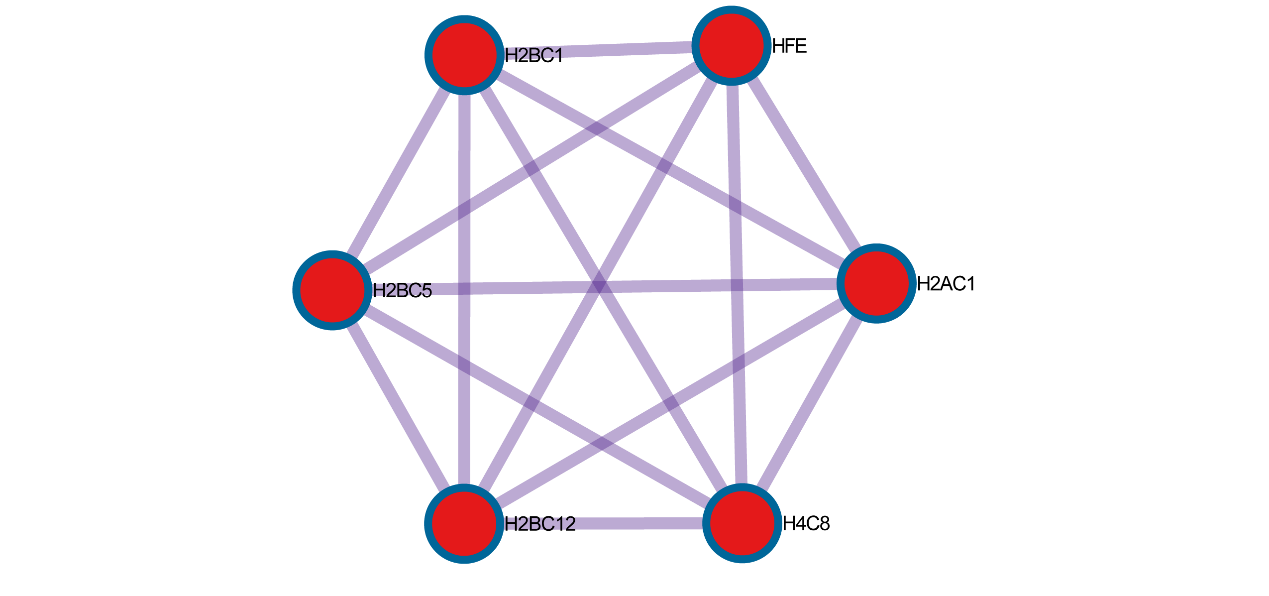


1. Li X, Li T, Wang J, Dong G, Zhang M, Xu Z, et al. Higher blood urea nitrogen level is independently linked with the presence and severity of neonatal sepsis. Annals of medicine. 2021;53(1):2192-8.

2. Li X, Zheng R, Zhang T, Zeng Z, Li H, Liu J. Association between blood urea nitrogen and 30-day mortality in patients with sepsis: a retrospective analysis. Annals of palliative medicine. 2021;10(11):11653-63.

3. Harazim M, Tan K, Nalos M, Matejovic M. Blood urea nitrogen - independent marker of mortality in sepsis. Biomedical papers of the Medical Faculty of the University Palacky, Olomouc, Czechoslovakia. 2023;167(1):24-9.

4. Gao M, Zhang L, Liu Y, Yang M, Wang N, Wang K, et al. Use of blood urea nitrogen, creatinine, interleukin-6, granulocyte-macrophage colony stimulating factor in combination to predict the severity and outcome of abdominal sepsis in rats. Inflammation research : official journal of the European Histamine Research Society [et al]. 2012;61(8):889-97.

5. Xie T, Xin Q, Cao X, Chen R, Ren H, Liu C, et al. Clinical characteristics and construction of a predictive model for patients with sepsis related liver injury. Clinica chimica acta; international journal of clinical chemistry. 2022;537:80-6.

6. Wang Y, Xi W, Zhang X, Bi X, Liu B, Zheng X, et al. CTSB promotes sepsis-induced acute kidney injury through activating mitochondrial apoptosis pathway. Front Immunol. 2022;13:1053754.

7. Cai S, Wang Q, Chen C, Guo C, Zheng L, Yuan M. Association between blood urea nitrogen to serum albumin ratio and in-hospital mortality of patients with sepsis in intensive care: A retrospective analysis of the fourth-generation Medical Information Mart for Intensive Care database. Front Nutr. 2022;9:967332.

8. Yoon J, Kym D, Hur J, Park J, Kim M, Cho YS, et al. The clinical differentiation of blood culture-positive and -negative sepsis in burn patients: a retrospective cohort study. Burns & trauma. 2023;11:tkad031.

9. Yu B, Chen M, Zhang Y, Cao Y, Yang J, Wei B, et al. Diagnostic and Prognostic Value of Interleukin-6 in Emergency Department Sepsis Patients. Infection and drug resistance. 2022;15:5557-66.

10. Jin L, Liao W, Zhou X, Wang Y, Qian J. Hydrocortisone alleviates sepsis-induced acute kidney injury through HSF-1-mediated transcriptional suppression of XPO1. Tissue & cell. 2022;79:101915.

11. Li JL, Li G, Jing XZ, Li YF, Ye QY, Jia HH, et al. Assessment of clinical sepsis-associated biomarkers in a septic mouse model. The Journal of international medical research. 2018;46(6):2410-22.

12. Li J, Wang Y, Luo J, Yin Z, Huang W, Zhang J. Development and validation of a nomogram for predicting sepsis in patients with pyogenic liver abscess. Sci Rep. 2023;13(1):10849.

13. Guarracino F, Bertini P, Pinsky MR. Cardiovascular determinants of resuscitation from sepsis and septic shock. Critical care (London, England). 2019;23(1):118.

14. Njim T, Dondorp A, Mukaka M, Ohuma EO. Identifying risk factors for the development of sepsis during adult severe malaria. Malaria journal. 2018;17(1):278.

15. Craciun FL, Iskander KN, Chiswick EL, Stepien DM, Henderson JM, Remick DG. Early murine polymicrobial sepsis predominantly causes renal injury. Shock (Augusta, Ga). 2014;41(2):97-103.

16. Kozyrakis D, Kratiras Z, Soukias G, Chatzistamou SE, Zarkadas A, Perikleous S, et al. Clinical Outcome and Prognostic Factors of Sepsis, Septic Shock and Prolonged Hospitalization, of Patients Presented with Acute Obstructive Pyelonephritis. Journal of endourology. 2020;34(4):516-22.

17. Abumayyaleh M, Nuñez-Gil IJ, El-Battrawy I, Estrada V, Becerra-Muñoz VM, Uribarri A, et al. Sepsis of Patients Infected by SARS-CoV-2: Real-World Experience From the International HOPE-COVID-19-Registry and Validation of HOPE Sepsis Score. Frontiers in medicine. 2021;8:728102.

18. Vizcarra-Jiménez D, Copaja-Corzo C, Hueda-Zavaleta M, Parihuana-Travezaño EG, Gutierrez-Flores M, Rivarola-Hidalgo M, et al. Predictors of Death in Patients with Neonatal Sepsis in a Peruvian Hospital. Tropical medicine and infectious disease. 2022;7(11).

19. Peng X, Chen C, Chen J, Wang Y, Yang D, Ma C, et al. Tree-based, two-stage risk factor analysis for postoperative sepsis based on Sepsis-3 criteria in elderly patients: A retrospective cohort study. Frontiers in public health. 2022;10:1006955.

20. Xiao Y, Yan X, Shen L, Wang Q, Li F, Yang D, et al. Evaluation of qSOFA score, and conjugated bilirubin and creatinine levels for predicting 28-day mortality in patients with sepsis. Experimental and therapeutic medicine. 2022;24(1):447.

21. Knox DB, Lanspa MJ, Kuttler KG, Brewer SC, Brown SM. Phenotypic clusters within sepsis-associated multiple organ dysfunction syndrome. Intensive Care Med. 2015;41(5):814-22.

22. Bai Y, Xia J, Huang X, Chen S, Zhan Q. Using machine learning for the early prediction of sepsis-associated ARDS in the ICU and identification of clinical phenotypes with differential responses to treatment. Frontiers in physiology. 2022;13:1050849.

23. Jazayeri A, Yang CC, Capan M. Frequent temporal patterns of physiological and biological biomarkers and their evolution in sepsis. Artificial intelligence in medicine. 2023;143:102576.

24. Xie C, Liu L, Wang Z, Xie H, Feng Y, Suo J, et al. Icariin Improves Sepsis-Induced Mortality and Acute Kidney Injury. Pharmacology. 2018;102(3-4):196-205.

25. Ou SM, Lee KH, Tsai MT, Tseng WC, Chu YC, Tarng DC. Sepsis and the Risks of Long-Term Renal Adverse Outcomes in Patients With Chronic Kidney Disease. Frontiers in medicine. 2022;9:809292.

26. Karamnov S, Brovman EY, Greco KJ, Urman RD. Risk Factors and Outcomes Associated With Sepsis After Coronary Artery Bypass and Open Heart Valve Surgeries. Seminars in cardiothoracic and vascular anesthesia. 2018;22(4):359-68.

27. Wang HE, Gamboa C, Warnock DG, Muntner P. Chronic kidney disease and risk of death from infection. American journal of nephrology. 2011;34(4):330-6.

28. Powell TC, Powell SL, Allen BK, Griffin RL, Warnock DG, Wang HE. Association of inflammatory and endothelial cell activation biomarkers with acute kidney injury after sepsis. SpringerPlus. 2014;3:207.

29. Yang HY, Hsu YO, Lee TH, Wu CY, Tsai CY, Chou LF, et al. Reduced Risk of Sepsis and Related Mortality in Chronic Kidney Disease Patients on Xanthine Oxidase Inhibitors: A National Cohort Study. Frontiers in medicine. 2021;8:818132.

30. Rodríguez-Núñez A, Cid E, Rodríguez-García J, Camiña F, Rodríguez-Segade S, Castro-Gago M. Concentrations of nucleotides, nucleosides, purine bases, oxypurines, uric acid, and neuron-specific enolase in the cerebrospinal fluid of children with sepsis. Journal of child neurology. 2001;16(9):704-6.

31. Mishra J, Jatav JK. To Study the Correlation or Association of Serum Uric Acid Level with Morbidities and Mortality in Sepsis Patient and its Prognostic Significance. The Journal of the Association of Physicians of India. 2022;70(4):11-2.

32. Liu S, Zhong Z, Liu F. Prognostic value of hyperuricemia for patients with sepsis in the intensive care unit. Sci Rep. 2022;12(1):1070.

33. Kir E, Güven Atici A, Güllü YT, Köksal N, Tunçez İ H. The relationship between serum uric acid level and uric acid/creatinine ratio with chronic obstructive pulmonary disease severity (stable or acute exacerbation) and the development of cor pulmonale. International journal of clinical practice. 2021;75(8):e14303.

34. Wang X, Wang Y, Antony V, Sun H, Liang G. Metabolism-Associated Molecular Patterns (MAMPs). Trends in endocrinology and metabolism: TEM. 2020;31(10):712-24.

35. Sreekanth, Maldar A. Hyperuricemia as an Early Marker in Predicting Mortality and Morbidity in Patients with Sepsis. The Journal of the Association of Physicians of India. 2022;70(4):11-2.

36. Aydın B, Dilli D, Zenciroğlu A, Karadağ N, Beken S, Okumuş N. Mean platelet volume and uric acid levels in neonatal sepsis. Indian journal of pediatrics. 2014;81(12):1342-6.

37. Akbar SR, Long DM, Hussain K, Alhajhusain A, Ahmed US, Iqbal HI, et al. Hyperuricemia: An Early Marker for Severity of Illness in Sepsis. International journal of nephrology. 2015;2015:301021.

38. Ganpule AP, Naveen Kumar Reddy M, Sudharsan SB, Shah SB, Sabnis RB, Desai MR. Multitract percutaneous nephrolithotomy in staghorn calculus. Asian journal of urology. 2020;7(2):94-101.

39. Bhanot R, Pietropaolo A, Tokas T, Kallidonis P, Skolarikos A, Keller EX, et al. Predictors and Strategies to Avoid Mortality Following Ureteroscopy for Stone Disease: A Systematic Review from European Association of Urologists Sections of Urolithiasis (EULIS) and Uro-technology (ESUT). European urology focus. 2022;8(2):598-607.

40. Wang J, Mi Y, Wu S, Shao H, Zhu L, Dai F. Impact Factors and an Efficient Nomogram for Predicting the Occurrence of Sepsis after Percutaneous Nephrolithotomy. BioMed research international. 2020;2020:6081768.

41. Chen X, Li S, Shi C, Zhang W, Liu Z, Jiang J, et al. Risk factors and predictors of urogenous sepsis after percutaneous nephrolithotomy for idiopathic calcium oxalate nephrolithiasis. Translational andrology and urology. 2023;12(6):1002-15.

42. Gao X, Lu C, Xie F, Li L, Liu M, Fang Z, et al. Risk factors for sepsis in patients with struvite stones following percutaneous nephrolithotomy. World journal of urology. 2020;38(1):219-29.

43. Taskin G, Sekerci CA, Tanidir Y, Cam K. The Significance of Asymptomatic Kidney Stones as a Predictive Factor for Sepsis in Critically Ill Older Adults. Puerto Rico health sciences journal. 2021;40(1):33-7.
